# Selective Involvement of a Subset of Spinal Dorsal Horn Neurons Operated by a Prodynorphin Promoter in Aβ Fiber-Mediated Neuropathic Allodynia-Like Behavioral Responses in Rats

**DOI:** 10.3389/fnmol.2022.911122

**Published:** 2022-06-23

**Authors:** Tadayuki Ishibashi, Yu Yoshikawa, Daichi Sueto, Ryoichi Tashima, Hidetoshi Tozaki-Saitoh, Keisuke Koga, Ken Yamaura, Makoto Tsuda

**Affiliations:** ^1^Department of Molecular and System Pharmacology, Graduate School of Pharmaceutical Sciences, Kyushu University, Fukuoka, Japan; ^2^Department of Anesthesiology and Critical Care Medicine, Graduate School of Medical Sciences, Kyushu University, Fukuoka, Japan; ^3^Department of Pharmaceutical Sciences, International University of Health and Welfare, Fukuoka, Japan; ^4^Department of Neurophysiology, Hyogo College of Medicine, Nishinomiya, Japan; ^5^Kyushu University Institute for Advanced Study, Fukuoka, Japan

**Keywords:** neuropathic mechanical allodynia, spinal dorsal horn, inhibitory neurons, primary afferent Aβ fibers, rat

## Abstract

Mechanical allodynia (pain produced by innocuous stimuli such as touch) is the main symptom of neuropathic pain. Its underlying mechanism remains to be elucidated, but peripheral nerve injury (PNI)-induced malfunction of neuronal circuits in the central nervous system, including the spinal dorsal horn (SDH), is thought to be involved in touch-pain conversion. Here, we found that intra-SDH injection of adeno-associated viral vectors including a prodynorphin promoter (AAV-PdynP) captured a subset of neurons that were mainly located in the superficial laminae, including lamina I, and exhibited mostly inhibitory characteristics. Using transgenic rats that enable optogenetic stimulation of touch-sensing Aβ fibers, we found that the light-evoked paw withdrawal behavior and aversive responses after PNI were attenuated by selective ablation of AAV-PdynP-captured SDH neurons. Notably, the ablation had no effect on withdrawal behavior from von Frey filaments. Furthermore, Aβ fiber stimulation did not excite AAV-PdynP^+^ SDH neurons under normal conditions, but after PNI, this induced excitation, possibly due to enhanced Aβ fiber-evoked excitatory synaptic inputs and elevated resting membrane potentials of these neurons. Moreover, the chemogenetic silencing of AAV-PdynP^+^ neurons of PNI rats attenuated the Aβ fiber-evoked paw withdrawal behavior and c-FOS expression in superficial SDH neurons. Our findings suggest that PNI renders AAV-PdynP-captured neurons excitable to Aβ fiber stimulation, which selectively contributes to the conversion of Aβ fiber-mediated touch signal to nociceptive. Thus, reducing the excitability of AAV-PdynP-captured neurons may be a new option for the treatment of neuropathic allodynia.

## Introduction

Neuropathic pain is a highly debilitating chronic pain condition that occurs following nerve damage to the somatosensory system as a consequence of compression by cancer, chemotherapy, diabetes, herpes zoster, or traumatic nerve injury (Colloca et al., 2017; Cohen et al., 2021). A hallmark symptom of neuropathic pain is mechanical allodynia, pain caused by innocuous mechanical stimuli. Because most existing analgesics are ineffective at treating neuropathic pain (Colloca et al., 2017), elucidating the mechanism of neuropathic pain and developing new therapeutics are important clinical challenges.

Somatosensory information from the periphery is conveyed to the spinal dorsal horn (SDH) *via* primary afferent neurons, processed, and transmitted to the brain (Todd, 2010). Among primary afferent neurons, low-threshold mechanoreceptors (LTMRs), such as Aβ fibers, are activated by innocuous mechanical stimuli (Abraira and Ginty, 2013) and under pathological conditions, such peripheral nerve injuries (PNIs) are implicated in mechanical allodynia (Xu et al., 2015). However, the mechanism for how touch information is converted to pain is not fully understood. Previous studies have identified specific subsets of neurons in the SDH as crucial for mechanosensory processing in the SDH (Duan et al., 2014; Foster et al., 2015; Peirs et al., 2015, 2021; Petitjean et al., 2015; Cui et al., 2016; Cheng et al., 2017). Among these subsets, ablation and acute silencing of the spinal inhibitory interneuron subset that expresses Cre recombinase in a *Prodynorphin* (*Pdyn*)*-Cre* mouse line induced behavioral hypersensitivity to mechanical stimuli by von Frey filaments and a paintbrush to the plantar skin (Duan et al., 2014; Zhang et al., 2018), suggesting that these neurons may play a role in neuropathic mechanical allodynia. However, the role of *Pdyn*-expressing SDH neurons remains controversial. It has been reported that loss of *Pdyn*-expressing neurons in mice lacking the transcription factor BHLHB5 did not affect mechanosensory behavioral responses (although these mice did exhibit spontaneous scratching behavior) (Ross et al., 2010; Kardon et al., 2014), and that activation of adult *Pdyn*−*Cre*^+^ SDH neurons elicits mechanical hypersensitivity (Huang et al., 2018). Thus, the role of *Pdyn*-expressing neurons in mechanosensory information processing, especially Aβ fiber-induced neuropathic allodynia, is not fully understood.

To investigate this, we used two tools. One was a transgenic rat line (W-TChR2V4) that expresses channelrhodopsin-2 (ChR2) at the nerve endings of touch-sensing Aβ fibers (Ji et al., 2012; Tashima et al., 2018). These rats allow us to selectively stimulate Aβ fibers by light illumination to the plantar skin, and our previous study showed that these rats exhibited light-evoked withdrawal behavior and aversive responses (resembling neuropathic allodynia) (Tashima et al., 2018, 2021). The other tool was an adeno-associated viral vector incorporating a rat *Pdyn* promoter (AAV-PdynP) used to visualize and operate PDYN-expressing SDH neurons in rats. Using these tools, we demonstrated that AAV-PdynP-captured SDH neurons selectively contribute to the PNI-induced mechanical allodynia-like behavioral responses evoked by optogenetic Aβ fiber stimulation but not by von Frey filaments.

## Materials and Methods

### Ethical Approval

All animal experiments were conducted according to the national and international guidelines contained in the “Act on Welfare and Management of Animals” (Ministry of Environment of Japan) and “Regulation of Laboratory Animals” (Kyushu University) and under the protocols approved by the Institutional Animal Care and Use Committee review panels at Kyushu University. All experimental procedures minimized the number and suffering of the animals. In this study, important adverse events were not observed in each experimental group.

### Animals

W-Tg (Thy1-COP4/YFP^*^)4Jfhy (W-TChR2V4: NBRPRat No. 0685) rats (Tomita et al., 2009; Ji et al., 2012) were supplied by the National BioResource Project—Rat, Kyoto University (Kyoto, Japan). All male W-TChR2V4 rats were aged 4–5 weeks at the start of each experiment and were housed in an individual cage at a temperature of 22 ± 1°C with a 12-h light-dark cycle (light from 8:00 to 20:00), and fed food and water *ad libitum*. W-TChR2V4 rats were randomly allocated to each experimental group.

### Recombinant AAV (rAAV) Vector Production

To produce a rAAV vector for *Pdyn* promoter-dependent gene transduction, a vector containing a *Pdyn* promoter (NCBI GenBank: NC_051338.1 and NM_019374.3; 2019 bp; −1846– +165 [+1= transcription start site]) was generated from pZac2.1 by substituting the CMV promoter with the *Pdyn* promoter. We then cloned tdTomato (tdT), diphtheria toxin receptors (DTR) (kindly provided by Prof. Kenji Kohno, Nara Institute of Science and Technology), HAtag-fused human muscarinic Gi-protein-coupled receptor (hM4Di; amplified from addgene plasmid #45548), enhanced green fluorescent protein (EGFP), and mCherry into the above-modified pZac2.1 to generate pZac2.1-PdynP-tdT-WPRE, pZac2.1-PdynP-DTR-EGFP-WPRE, and pZac2.1-PdynP-hM4Di-2A-mCherry-WPRE. These rAAV vectors were produced from human embryonic kidney 293T (HEK293T) cells with triple transfection [pZac, cis plasmid; pAAV2/9, trans plasmid; and pAd DeltaF6, adenoviral helper plasmid (all plasmids were purchased from the University of Pennsylvania Gene Therapy Program Vector Core)] and purified by two cesium chloride density gradient purification steps. The vector was dialyzed against phosphate-buffered saline (PBS) containing 0.001% (v/v) Pluronic-F68 using Amicon Ultra 100 K filter units (Millipore, Darmstadt, Germany). The genome titer of rAAV was determined by Pico Green fluorometric reagent (Molecular Probes, OR, USA) following denaturation of the AAV particle. Vectors were stored in aliquots at −80°C until use.

### Intra-SDH Injection of rAAV Vectors

Under isoflurane (2%) anesthesia, rats were intraperitoneally (i.p.) injected with pentobarbital (65 mg/kg). The skin was incised at Th12–L3 and custom-made clamps were attached to the caudal side of the vertebral column. Paraspinal muscles around the left side of the interspace between Th13 and L1 vertebrae were removed, and the dura mater and the arachnoid membrane were carefully incised using the tip of a 30G needle to make a small window. Then, we inserted the microcapillary backfilled with rAAV solution into the SDH (500 μm lateral from the midline and 150 μm in depth from the surface of the dorsal root entry zone) and microinjected 800 nl rAAV solution (3 × 10^12^ GC/ml) using FemtoJet Express (Eppendorf, Hamburg, Germany) (Kohro et al., 2015; Tashima et al., 2021). After microinjection, the inserted microcapillary was removed from the SDH, the skin was sutured with 3–0 silk, and rats were kept under a heating light source until recovery.

### Immunohistochemistry

Rats were anesthetized with pentobarbital (100 mg/kg, i.p.) and perfused transcardially with 100 ml of PBS, followed by 250 ml ice-cold 4% paraformaldehyde/PBS. The L4 segments of the spinal cord were removed, postfixed in the same fixative for 3 h at 4°C, placed in 30% sucrose solution for 24–48 h at 4°C, embedded by Tissue-Tek O.C.T. Compound (Sakura Finetek Japan, Tokyo, Japan), and stored at −80°C before use. Transverse sections of the L4 spinal cord (30 μm) were made from the cryoprotected samples using a cryostat (Leica CM1100) and were immunostained by the free-floating method as described previously (Tsuda et al., 2011; Tashima et al., 2018). L4 spinal sections were incubated in blocking solution for 2 h and followed by the primary antibodies: polyclonal rabbit anti-neurokinin 1 receptor (NK1R; 1:10000; S8305, Sigma-Aldrich, MO, USA), isolectin B4 (IB4), biotin-conjugate (1:1000; I21414, Thermo Fisher Scientific, MA, USA), polyclonal guinea pig anti-prodynorphin (PDYN; 1:200; AB5519, Millipore, MA, USA), polyclonal goat anti-paired box 2 (PAX2; 1:500; AF3364, R&D Systems, MN, USA), polyclonal guinea pig anti-vesicular glutamate transporter 2 (VGLUT2; 1:1000; AB2251-I, Millipore, MA, USA), polyclonal goat anti-DTR (1:500; AF-259, R&D Systems, MN, USA), and monoclonal rat anti-mCherry (1:2000; M11217, Thermo Fisher Scientific, MO, USA) for 48 h at 4°C. The sections were washed and incubated with secondary antibodies conjugated with Alexa Fluor 405, 488, and 546 (1:1000; A11008, A11055, A11056, A11073, 20373-500UL, 706-546-148, 711-476-152, 712-165-153 and S32351, Molecular, Probes) for 3 h. The sections were then analyzed by a confocal microscope (LSM700, Zeiss, Oberkochen, Germany). For quantification of co-expression of AAV-PdynP^+^ neurons and PDYN, we performed z-stack imaging to reliably identify AAV-PdynP^+^ neurons positive for PDYN immunofluorescence in their perikaryal cytoplasm (Sardella et al., 2011). For c-FOS immunostaining in the SDH, the W-TChR2V4 rats were habituated for 10 min under no anesthesia, and the plantar surface of the hindpaw of the rats was simulated for 10 min with blue light (frequency, 5 Hz; interval, 500 ms) without anesthetics. At 2 h after optical stimulation, the rats were deeply anesthetized with pentobarbital (100 mg/kg, i.p.) and fixed as described above, and the L4 spinal cord was obtained. Transverse spinal cord (30 μm) sections were stained by primary antibodies: monoclonal rabbit anti-c-FOS (1:1,000; 2,250, Cell Signaling Technology, MA, USA). For quantification, three to four sections from the L4 spinal cord segments were randomly selected from each rat, and the number of c-FOS^+^ neurons in the superficial laminae I–II was counted with ImageJ software (http://rsbweb.nih.gov/ij/). For counting c-FOS^+^ neurons in experiments related to **Figure 5**, we counted the c-FOS^+^ neurons in the superficial laminae (laminae I–II) without the Venus fluorescence expressed from Aβ fibers in the W-TChR2V4 rats (Tashima et al., 2018, 2021). In our confocal microscope system, there were three channels for detecting fluorescent colors; green for Venus expressed in W-TChR2V4 rats (Tomita et al., 2009), red for tdT or mCherry expressed in AAV-PdynP^+^ neurons, and blue for c-FOS immunostaining (blue was changed to green in **Figure 5C** to improve visibility). Thus, there was no additional channel available to detect additional colors. Therefore, we could not visualize the lamina region using specific markers.

### Neuropathic Pain Models

We used the spinal nerve injury model with some modifications (Kim and Chung, 1992; Tsuda et al., 2011). In brief, under isoflurane (2%) anesthesia, the left fifth lumbar (L5) spinal nerve was tightly ligated with 5–0 silk and cut just distal to the ligature. Sham operation was performed by exposing the transverse process of the lumbar vertebra without removing the process to keep the L5 spinal nerve intact. The wound and the surrounding skin were sutured with 3–0 silk.

### Whole-Cell Path-Clamp Recording

As previously described (Tashima et al., 2018, 2021), under anesthesia with urethane (1.2–1.5 g/kg, i.p.), the lumbosacral spinal cord was removed from wild-type (WT) or W-TChR2V4 rats and placed into a cold, high-sucrose, artificial cerebrospinal fluid (ACSF) (250 mM sucrose, 2.5 mM KCl, 2 mM CaCl_2_, 2 mM MgCl_2_, 1.2 mM NaH_2_PO_4_, 25 mM NaHCO_3_, and 11 mM glucose). A parasagittal spinal cord slice (300–350 μm thick) with an attached L4 dorsal root was made using a vibrating microtome (VT1200, Leica), and the slices were maintained in oxygenated ACSF solution (125 mM NaCl, 2.5 mM KCl, 2 mM CaCl_2_, 1 mM 5 MgCl_2_, 1.25 mM NaH_2_PO_4_, 26 mM NaHCO_3_, and 20 mM glucose) at room temperature (22–25°C) for at least 30 min. The spinal cord slice was then put into a recording chamber where oxygenated ACSF solution (26–28°C) was continuously superfused at a flow rate of 4 to 7 ml/min. SDH neurons were visualized with an upright microscope equipped with infrared differential interference contrast Nomarski (FN1, Nikon, Tokyo, Japan). The cellular location was confirmed by immunohistochemistry, in which cells were loaded with 0.4% Neurobiotin via a patch pipette. The patch pipettes were filled with an internal solution (125 mM K-gluconate, 10 mM KCl, 0.5 mM EGTA, 10 mM HEPES, 4 mM ATP-Mg, 0.3 mM NaGTP, 10 mM phosphocreatine, 0.4% Neurobiotin, pH 7.28 adjusted with KOH). The pipette tip resistance was 4 to 7 MΩ. Synaptic currents were recorded using a computer-controlled amplifier (Axopatch 700B, Molecular Devices, CA, USA). The data were digitized with an analog-to-digital converter (Digidata 1550, Molecular Devices), stored on a personal computer using a data acquisition program (pCLAMP 10.4 acquisition software, Molecular Devices), and analyzed using a software package (Clampfit version 10.6, Molecular Devices). Excitatory postsynaptic currents (EPSCs) were recorded in the voltage-clamp mode at a holding potential of −70 mV, and the dorsal roots were stimulated with a suction electrode. An optical fiber with a 200-μm tip width was placed on the proximal part of the dorsal root. Optical stimuli (blue light intensity, 4 V; duration, 5 ms) were applied to the dorsal root for stimulating Aβ fibers as described previously (Tashima et al., 2021). Light-evoked EPSCs were considered to be monosynaptic responses if they did not exhibit failures upon repetitive stimulation at 1 Hz and if they exhibited a low jitter (<1 ms) upon stimulation at 0.1 Hz (Petreanu et al., 2007; Honsek et al., 2015). The membrane potentials were recorded in the current-clamp mode, and the firing patterns were determined by passing 1.0 s depolarizing current pulses through the recording electrode at the resting membrane potential.

### Light Illumination of the Hindpaw

According to our previous methods (Tashima et al., 2018, 2021), rats were placed on a transparent acrylic plate and habituated for 30–60 min to allow acclimatization to the new environment. The plantar surface of the hindpaw (touching the acrylic plate floor) was illuminated with a blue laser diode (COME2-LB473/532/100, Lucir, Osaka, Japan: wavelength, 470 nm; frequency, 5 Hz; interval, 10 s; 10 times per each ipsilateral and contralateral hindpaw) over the acrylic plate. The light power intensity (1 mV) was measured by a thermopile (COME2-LPM-NOVA, Lucir), which was a laser power meter with 1 mW at the skin. Withdrawal responses of the hindpaw to light illumination (0: no reaction, 1: mild movement without any lifting and flinching behaviors, 2: hindpaw lifting and flinching) were calculated from total scores of 10 times per hindpaw (Tashima et al., 2018, 2021). To investigate the light-induced responses of animals, we were careful to establish experimental conditions before light stimuli. First, the animals were awake. Second, both hindpaws were attached to the floor of the acrylic plate. Third, the animals were at rest without moving or walking.

### von Frey Test

This method used the von Frey test as previously described (Tashima et al., 2018, 2021). In brief, calibrated von Frey filaments (0.4–15 g, Stoelting, IL, USA) were applied to the plantar surface of the rat hindpaw, and the 50% paw withdrawal threshold was determined.

### Plantar Thermal Test

Noxious heat-evoked paw withdrawal response was detected by application of radiant heat to the plantar surface of hindpaw. The hindpaw was positioned at a heat source in an apparatus (Ugo Basile, Gemonio, Italy). The time taken for the rat to withdraw from heat stimuli was measured as the withdrawal latency. The measurement was conducted three times on the ipsilateral and contralateral sides.

### Place Aversion

This test was performed in accordance with a method described previously (Tashima et al., 2018, 2021). W-TChR2V4 rats were placed in a two-chambered box (45 cm width, 10 cm depth, 40 cm height) with black and white compartments and were allowed to freely explore the two chambers for 15 min (acclimatization to the new environment). At first, the rat location was continuously monitored and analyzed for time spent in each of the chambers without light stimulation. After that, the rat was brought back to the home cage for 15 min, and it was placed in the two-chambered box again; then the test session was started. During the test session (20 min), blue light stimuli (wavelength, 470 nm; frequency, 5 Hz; interval; 500 ms; light illumination interval, 5 s) were applied to the plantar skin of the left hindpaw of each rat with or without DTX through a transparent acrylic plate floor, only when the testing rat was in the black compartment. The time spent in each compartment was measured for 20 min. To assess the aversive response to the light side (black compartment), we calculated the percentage of time spent in the black compartment.

### Statistical Analysis

All data are shown as the mean ± SEM. Statistical analyses of the results were conducted with unpaired *t*-test (**Figures 2C**, **4E**), one-way ANOVA with *post-hoc* Tukey's multiple comparison test (**Figure 2D**), one-way ANOVA with *post-hoc* Bonferroni multiple comparison tests (**Figure 5D**), two-way factorial ANOVA with *post-hoc* Tukey's multiple comparison test (**Figure 3B**), two-way ANOVA with *post-hoc* Bonferroni multiple comparison tests (**Figure 5B**), two-way repeated-measures ANOVA with *post-hoc* Bonferroni multiple comparison tests (**Figures 3A**, **4G**), Mann–Whitney *U* test (**Figure 4C**), Fisher's exact test (**Figure 4D**), and linear regression and individual regression coefficients (**Figure 4F**) using the GraphPad Prism 7.01 software (GraphPad Software Inc., CA, USA). *P*-values are indicated as ^*^P < 0.05, ^**^P < 0.01, ^***^P < 0.001, and ^****^P < 0.0001.

## Results

### The Subset of SDH Neurons Captured by the AAV-PdynP Vector

**Figure 1:**
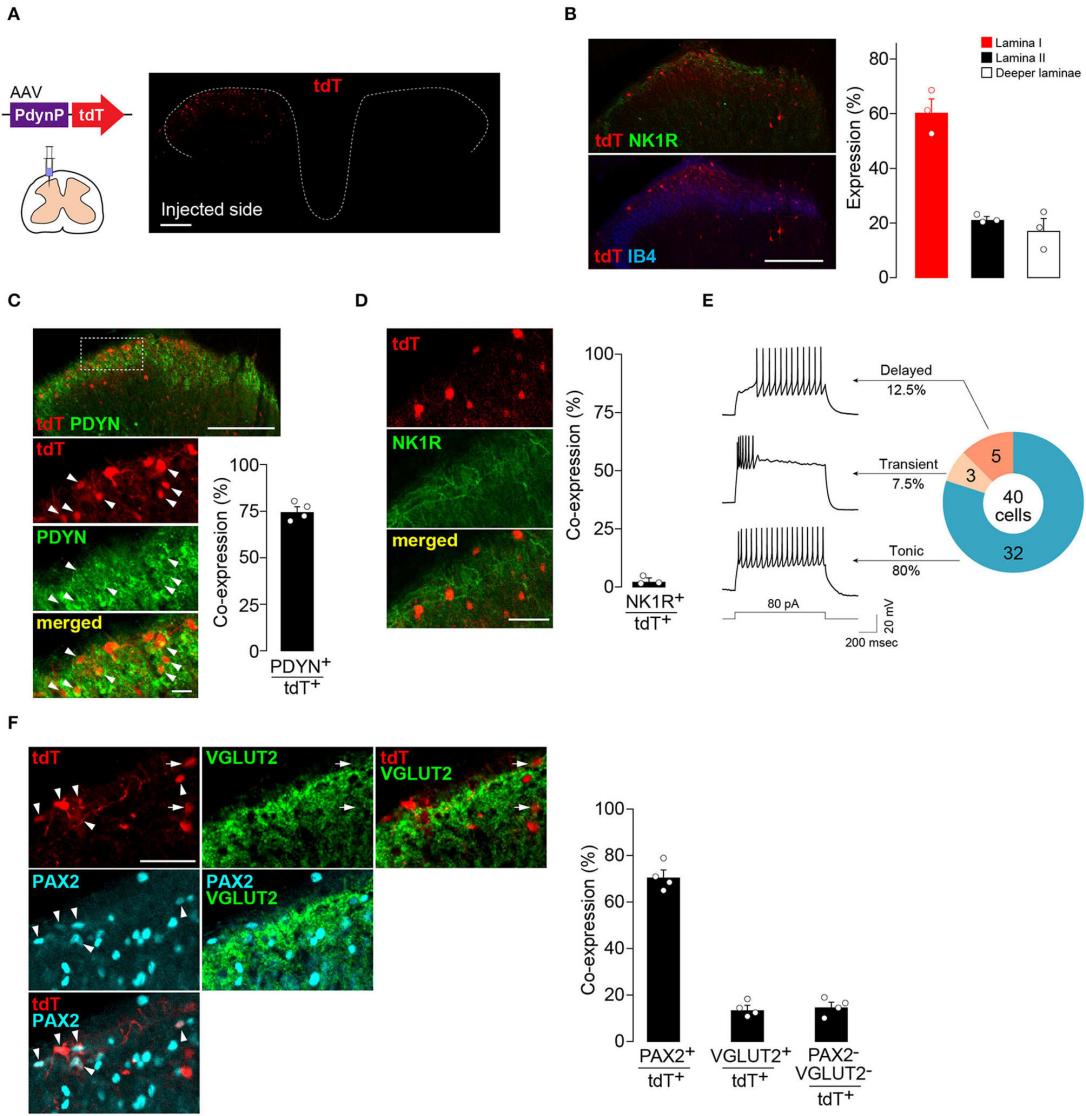
AAV-PdynP injected into rat spinal dorsal horn (SDH) captures a subset of inhibitory neurons. **(A)** tdTomato (tdT) expression in the fourth lumbar (L4) SDH at 4 weeks after microinjection of AAV-PdynP-tdT. Schematic illustration indicates intra-SDH microinjection of the AAV vector. Scale bar, 200 μm. **(B)** Immunolabeling of tdT^+^ cells (red) with lamina-selective markers (green, NK1R; blue, IB4) in the L4 SDH. Scale bar, 200 μm. Quantification of the distribution of tdT^+^ cells (*n* = 3 rats tested, 272 total tdT^+^ cells quantified). **(C)** Immunofluorescence of prodynorphin (PDYN; green) in tdT^+^ SDH neurons (red). Scale bar, 200 μm (above), 20 μm (below). Arrowheads indicate tdT^+^ PDYN^+^ neurons. The percentage of tdT^+^ PDYN^+^ neurons per total tdT^+^ neurons was quantified (*n* = 4 rats tested, 1,281 total tdT^+^ cells quantified). **(D)** No colocalization of tdT (red) with NK1R (green). Scale bar, 50 μm. The percentage of co-expressing neurons was quantified (*n* = 3 rats tested, 187 total tdT^+^ cells quantified). **(E)** Firing patterns of tdT^+^ SDH neurons, and the percentage of neurons displaying each pattern. APs were evoked by current injection (80 pA). **(F)** Colocalization of tdT (red) with PAX2 (blue) and VGLUT2 (green) in the superficial laminae. Arrowheads and arrows indicate tdT^+^PAX2^+^ and tdT^+^VGLUT2^+^ neurons, respectively. Scale bar, 50 μm. The percentage of co-expressing neurons was quantified (*n* = 4 rats tested, 456 total tdT^+^ cells quantified). Data shown as mean ± SEM.

First, to visualize and characterize the SDH neurons captured by AAV-PdynP, the vector incorporating the gene encoding tdTomato (tdT) was microinjected into the SDH of WT rats according to our previously established methods (Kohro et al., 2015; Tashima et al., 2021) (Figure 1A). Four weeks later, tdT fluorescence was observed in the injected side (ipsilateral side) of the SDH, but not in the contralateral side (Figure 1A). Immunohistochemical analysis using antibodies for neurokinin 1 receptor (NK1R) and isolectin B4 (IB4), makers for laminae I and IIi, respectively (Todd, 2010), revealed that tdT^+^ neurons were located predominantly in the superficial laminae and, to a lesser extent, in deeper laminae (Figure 1B). Furthermore, we confirmed that the majority of tdT^+^ neurons in the superficial laminae expressed PDYN (Figure 1C). There were PDYN^+^ neurons with and without tdT expression. In addition, almost no AAV-PdynP^+^ neurons in the superficial laminae had immunofluorescence for NK1R (Figure 1D), which is known to be expressed in brain-projecting neurons (Todd, 2010). These results indicate that our designed AAV-PdynP vector captures a subset of interneurons expressing PDYN in the rat SDH. Hereafter, we refer to this subset as AAV-PdynP^+^ neurons. AAV-PdynP^+^ SDH neurons in the superficial laminae were also electrophysiologically characterized using whole-cell patch-clamp recordings. Under a current clamp mode, injecting a depolarizing current at the resting membrane potential (RMP) induced a tonic firing pattern in 80% of tdT^+^ neurons and the remaining tdT^+^ neurons produced a delayed or transient firing pattern (Figure 1E). Moreover, we performed double immunostaining for PAX2 (as a marker of inhibitory neurons) (Cheng et al., 2005; Koga et al., 2017) and VGLUT2 (as a marker of excitatory neurons) (Todd, 2010) and quantified AAV-PdynP^+^ neurons with and without PAX2 and VGLUT2 expressions. In the superficial laminae, 70.9% of AAV-PdynP^+^ neurons were positive for PAX2 (Figure 1F), but the percentage of AAV-PdynP^+^ VGLUT2^+^ neurons was much lower (~14.0%). Together, these findings suggest that the AAV-PdynP-captured SDH neurons in rats are mostly inhibitory interneurons.

### The Role of AAV-PdynP^+^ SDH Interneurons in Somatosensory Behaviors Under Normal and Pathological Conditions

**Figure 2:**
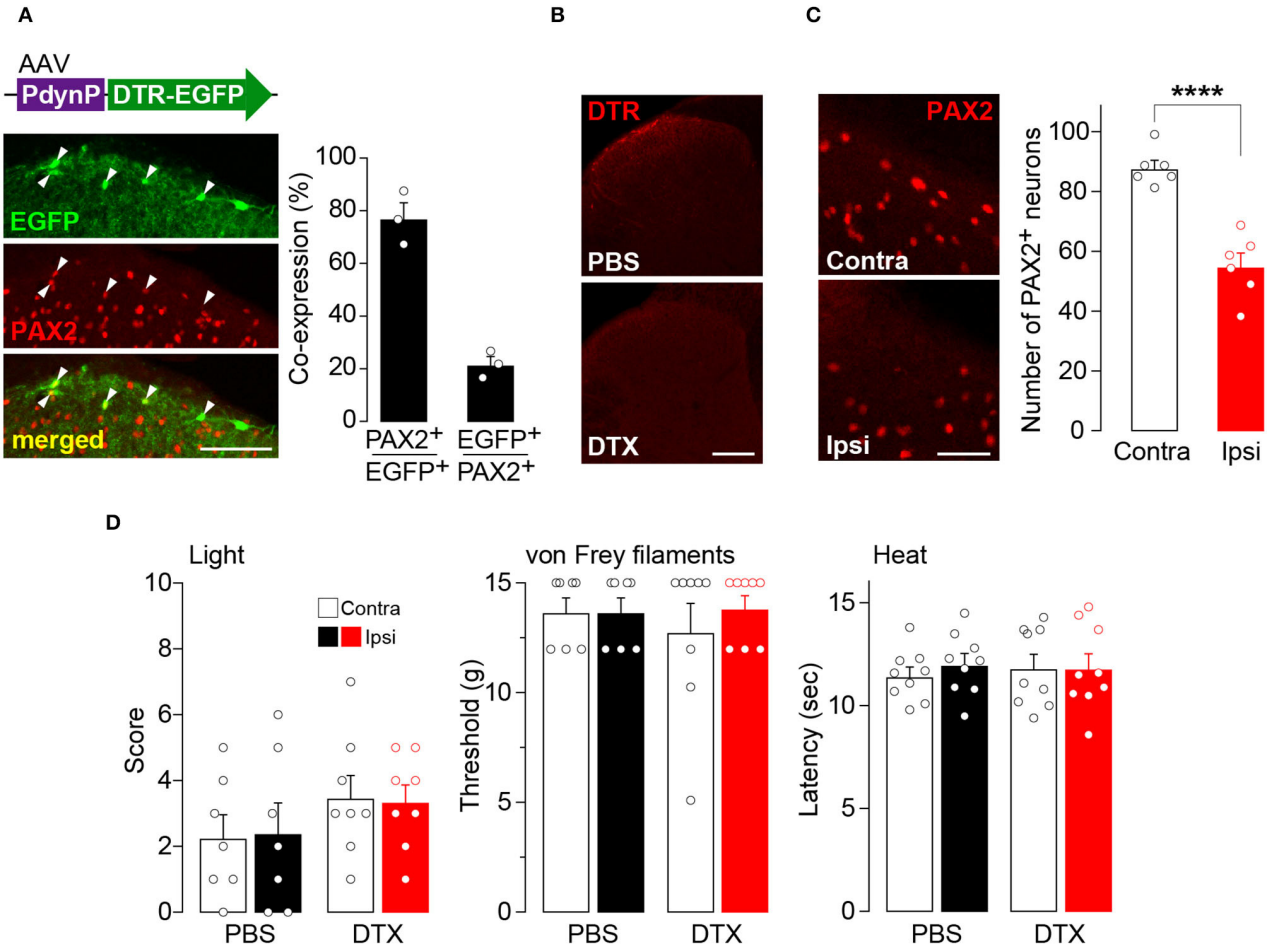
Ablation of AAV-PdynP^+^ spinal dorsal horn (SDH) neurons does not affect behavioral responses evoked by the optical stimulation of Aβ fibers, nociceptive mechanical force, and heat in rats. **(A)** Enhanced green fluorescent protein (EGFP) expression (green) in the L4 SDH at 4 weeks after intra-SDH microinjection of AAV-PdynP-DTR-EGFP (indicated by an upper schematic illustration). Colocalization of EGFP and PAX2 (red) in the SDH (indicated by arrowheads). Scale bar, 100 μm. The percentage of co-expressing neurons was quantified (*n* = 3 rats tested, 94 total EGFP^+^ cells quantified, and 339 total PAX2^+^ cells quantified). **(B)** Diphtheria toxin receptors (DTR) immunofluorescence in the SDH after administration of diphtheria toxin (DTX: 50 μg/kg, i.p., two injection 72 h apart) or PBS. Scale bar, 200 μm. **(C)** Immunohistochemical analysis and quantification of PAX2^+^ neurons in the superficial laminae of the ipsilateral and contralateral SDH at 4 weeks after DTX injection (*n* = 6 rats tested, PAX2^+^ neurons: 992 [ipsi] and 1574 [contra] cells quantified). Scale bar, 50 μm. ****P < 0.0001, Unpaired *t*-test. **(D)** Paw withdrawal behaviors by light (score), von Frey filaments (threshold), and heat (latency) at 4 weeks after DTX or PBS injection (*n* = 7–9 rats). Data shown as mean ± SEM.

To examine the role of AAV-PdynP^+^ neurons in somatosensory behavior, we employed a cell ablation approach (Saito et al., 2001). DTR was expressed in AAV-PdynP^+^ SDH neurons by microinjecting AAV-PdynP-DTR-EGFP into the SDH of W-TChR2V4 rats (Figure 2A). Consistent with the data in WT rats (Figure 1), the majority of EGFP^+^ neurons in W-TChR2V4 rats were also located in superficial laminae and were positive for PAX2 immunofluorescence. Four weeks after DTX treatment (50 μg/kg, i.p., two injections 72 h apart), DTR^+^ SDH neurons were ablated (Figure 2B), which resulted in a significant decrease in the number of PAX2^+^ interneurons in the AAV-injected SDH (Figure 2C). Behaviorally, the W-TChR2V4 rats with AAV-PdynP^+^ SDH neuron ablation did not change their hindpaw withdrawal responses from any stimuli including illuminating blue light (to stimulate Aβ fibers), applying von Frey filaments, or applying noxious heat to the plantar skin (Figure 2D). Behavioral responses to these stimuli in the contralateral hindpaw were also not changed. These findings indicate that under normal conditions, AAV-PdynP^+^ interneurons are not involved in behavioral responses evoked by the optical stimulation of Aβ fibers, as well as nociceptive mechanical force and heat.

**Figure 3:**
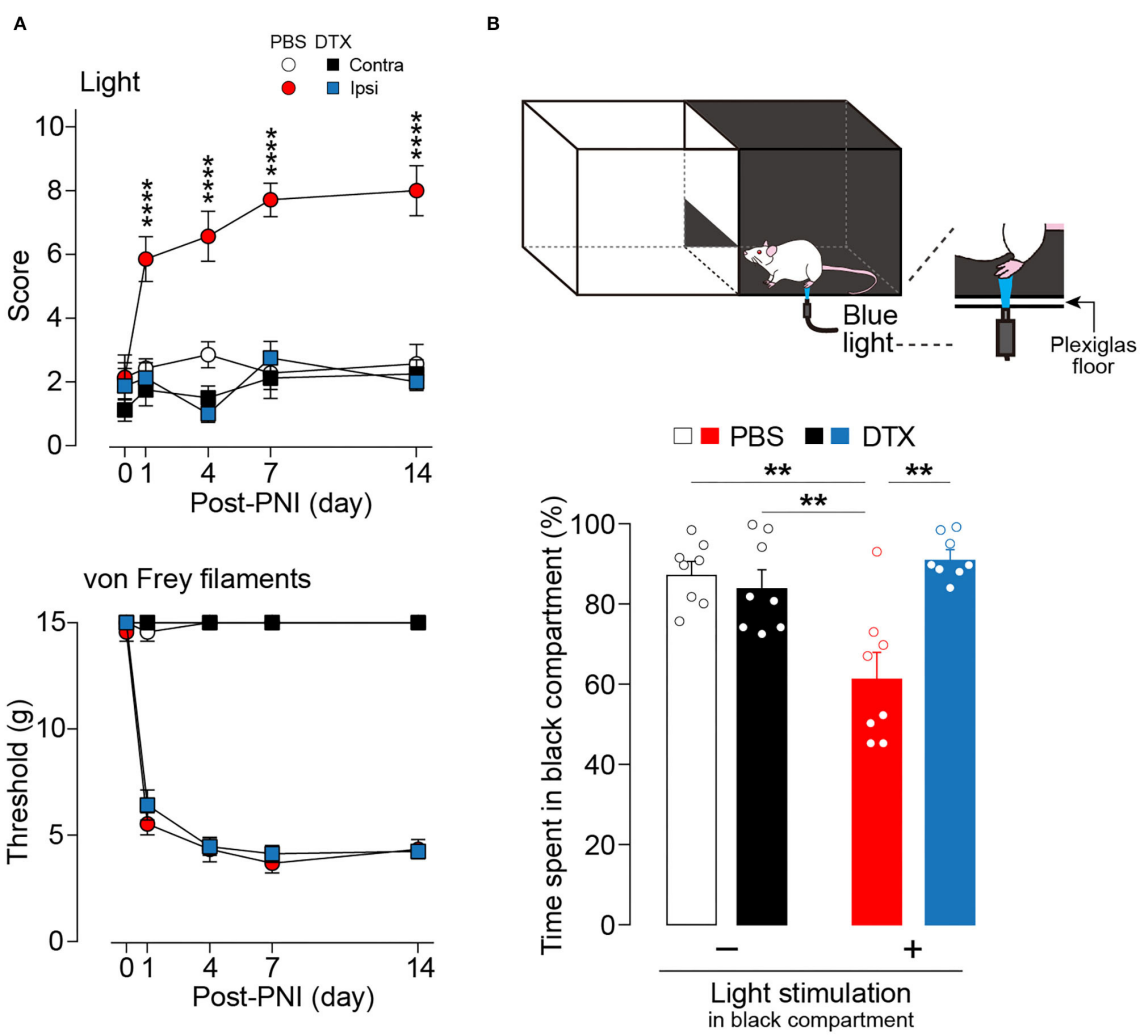
Ablation of AAV-PdynP^+^ spinal dorsal horn (SDH) neurons selectively attenuates optogenetic Aβ fiber-induced pain-like behavior after peripheral nerve injury (PNI) in rats. **(A)** Paw withdrawal responses to light and von Frey filaments in W-TChR2V4 rats with and without AAV-PdynP^+^ SDH neurons after PNI (*n* = 7–8 rats). ****P < 0.0001 vs. the ipsilateral side of DTX-treated rats, two-way repeated measures ANOVA with *post-hoc* Bonferroni multiple comparison test. **(B)** Schematic diagram for place aversion test. Rats received blue light to the plantar skin while in the black compartment. The percentage of time spent in black compartment with or without light stimulation 2 weeks after PNI in W-TChR2V4 rats with and without AAV-PdynP^+^ SDH neurons after PNI (*n* = 8). **P < 0.01, two-way factorial ANOVA with *post-hoc* Tukey's multiple comparison test. Data shown as mean ± SEM.

Next, the effect of the ablation of these neurons in a neuropathic pain model was examined. The increase in withdrawal responses evoked by light illumination following PNI was markedly suppressed by ablation of AAV-PdynP^+^ SDH neurons (Figure 3A). In contrast, the ablation did not affect PNI-induced behavioral hypersensitivity to mechanical stimuli with von Frey filaments. Furthermore, to evaluate the aversive response evoked by optical stimulation of Aβ fibers after PNI, we employed a real-time place aversion assay in which light was applied to the plantar skin only when the tested rat was in a black compartment (Figure 3B) (Tashima et al., 2018, 2021). After PNI, the PBS-treated W-TChR2V4 rats with AAV-PdynP-DTR-EGFP significantly decreased their time spent in the black side with light stimulation compared to rats without light. However, in AAV-PdynP^+^ neurons-ablated PNI rats, no change was observed, implying attenuation of optogenetic Aβ fiber-induced aversive response after PNI. These results suggest a critical role of AAV-PdynP^+^ SDH interneurons in Aβ fiber-induced neuropathic allodynia-like behavioral responses.

### Optogenetic Aβ Fiber-Induced Excitation of AAV-PdynP^+^ SDH Neurons After PNI

**Figure 4:**
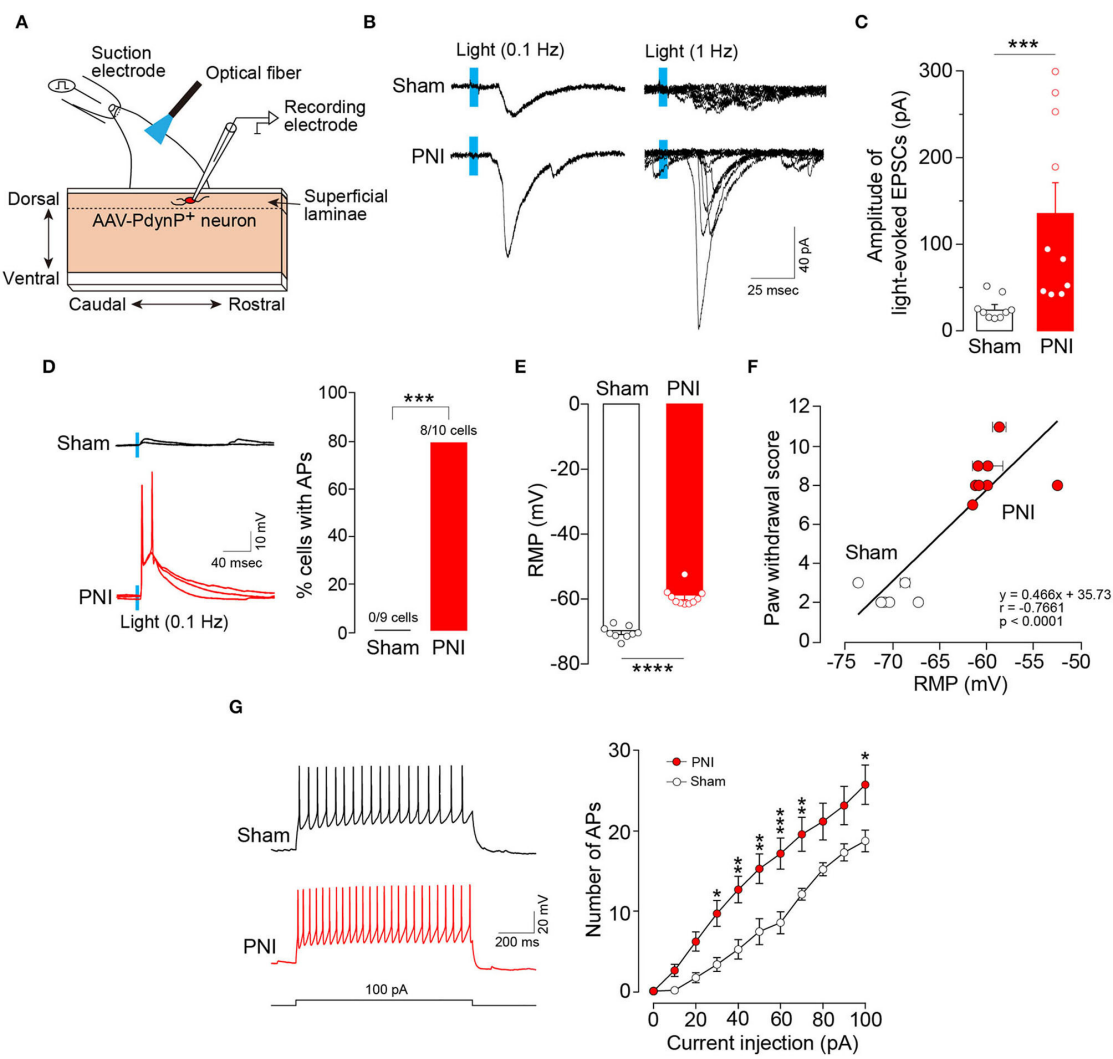
Excitability of AAV-PdynP^+^ neurons is enhanced after peripheral nerve injury (PNI). **(A)** Schematic diagram of whole-cell recording in AAV-PdynP^+^ neurons using a sagittal spinal cord slice of the L4 dorsal root from W-TChR2V4 rat. **(B)** Representative traces of excitatory postsynaptic currents (EPSCs) evoked by blue light (0.1 and 1 Hz) in AAV-PdynP^+^ neurons in spinal cord slices of rats with sham operation and PNI. **(C)** Quantitative analysis of the amplitude of light-evoked EPSCs of AAV-PdynP^+^ neurons in sham-operated and PNI rats (*n* = 9 neurons tested from 6 W-TChR2V4 rats with sham operation, *n* = 10 neurons tested from 8 W-TChR2V4 rats with PNI). ***P < 0.001, Mann–Whitney *U* test. **(D)** Representative traces of action potentials (APs) evoked by light (blue) of AAV-PdynP^+^ neurons. Percentage of AAV-PdynP^+^ neurons with or without APs was quantified. ***P < 0.001, Fisher's exact test. **(E)** Resting membrane potentials (RMPs: mV) of AAV-PdynP^+^ neurons in slices from sham-operated and PNI rats. ****P < 0.0001, unpaired *t* test. **(F)** Correlation plot showing the relationship between paw withdrawal score and RMPs of AAV-PdynP^+^ neurons in rats with sham operation and PNI (*n* = 6 W-TChR2V4 rats with sham operation, *n* = 8 W-TChR2V4 rats with PNI). Note that we recorded one neuron from one spinal cord slice from each animal in most cases, but in some cases, two or more neurons were recorded. Thus, some data were depicted with error bars. **(G)** Representative trace of APs evoked by injecting depolarizing currents (100 pA, 1 s) into AAV-PdynP^+^ neurons in spinal cord slices of rats with sham operation and PNI. The relationship between firing frequency and injected current of AAV-PdynP^+^ neurons (*n* = 9 neurons tested from 6 W-TChR2V4 rats with sham operation, *n* = 10 neurons tested from 8 W-TChR2V4 rats with PNI). *P < 0.05, **P < 0.01, and ***P < 0.001 vs. sham, two-way repeated measures ANOVA with *post-hoc* Bonferroni multiple comparison test. Data shown as mean ± SEM.

To examine the synaptic inputs from Aβ fibers to AAV-PdynP^+^ neurons, we used spinal cord slices of the L4 dorsal root of W-TChR2V4 rats and measured EPSCs in AAV-PdynP^+^ neurons after Aβ fiber stimulation by illuminating light on the dorsal root (Figure 4A). Optogenetic Aβ fiber stimulation evoked polysynaptic EPSCs (21/25 neurons). However, the amplitude of polysynaptic EPSCs in the AAV-PdynP^+^ neurons was relatively small (34.85 ± 6.75 pA, *n* = 21 neurons), and few (1/25 neuron tested) produced action potentials (APs) by optogenetic Aβ fiber stimulation. Similarly, AAV-PdynP^+^ neurons in sham-operated W-TChR2V4 rats also had small amplitudes in EPSCs and no APs by optogenetic Aβ fiber stimulation (Figures 4B–D) (*n* = 9 neurons tested from 6 W-TChR2V4 rats with sham operation). Contrarily, in spinal cord slices from W-TChR2V4 rats with PNI, the amplitudes of EPSCs in AAV-PdynP^+^ neurons by optogenetic Aβ fiber stimulation were markedly increased (Figures 4B,C) (*n* = 10 neurons tested from 8 W-TChR2V4 rats with PNI). Furthermore, 80% of these neurons exhibited APs in response to the optogenetic Aβ fiber stimulation (Figure 4D). Analyzing the electrophysiological properties under a current-clamp mode, we further found that the RMPs of AAV-PdynP^+^ neurons were significantly elevated in PNI rats compared to sham-operated rats (Figure 4E). There was also a significant correlation between the RMPs and the score of optogenetic Aβ fiber-induced paw withdrawal behavior obtained from rats with sham or PNI (Figure 4F) (*n* = 6 W-TChR2V4 rats with sham operation, *n* = 8 W-TChR2V4 rats with PNI). The number of APs evoked by current injection at RMP in AAV-PdynP^+^ neurons was significantly increased after PNI (Figure 4G). The firing patterns did not change after PNI; delayed firing was observed in one of 11 neurons tested, and other electrophysiological data in AAV-PdynP^+^ neurons were not altered by PNI (input resistance: sham, 393.37 ± 99.24 MΩ, PNI, 426.39 ± 70.43 MΩ; membrane capacitance: sham, 44.56 ± 5.20 pF, PNI, 44.99 ± 4.66 pF) (Supplemental Table 1). Collectively, these findings suggest that PNI renders AAV-PdynP^+^ neurons excitable to optogenetic Aβ fiber stimulation.

### Silencing AAV-PdynP^+^ SDH Neurons Attenuates Optogenetic Aβ Fiber-Induced Neuropathic Allodynia-Like Behavior

**Figure 5:**
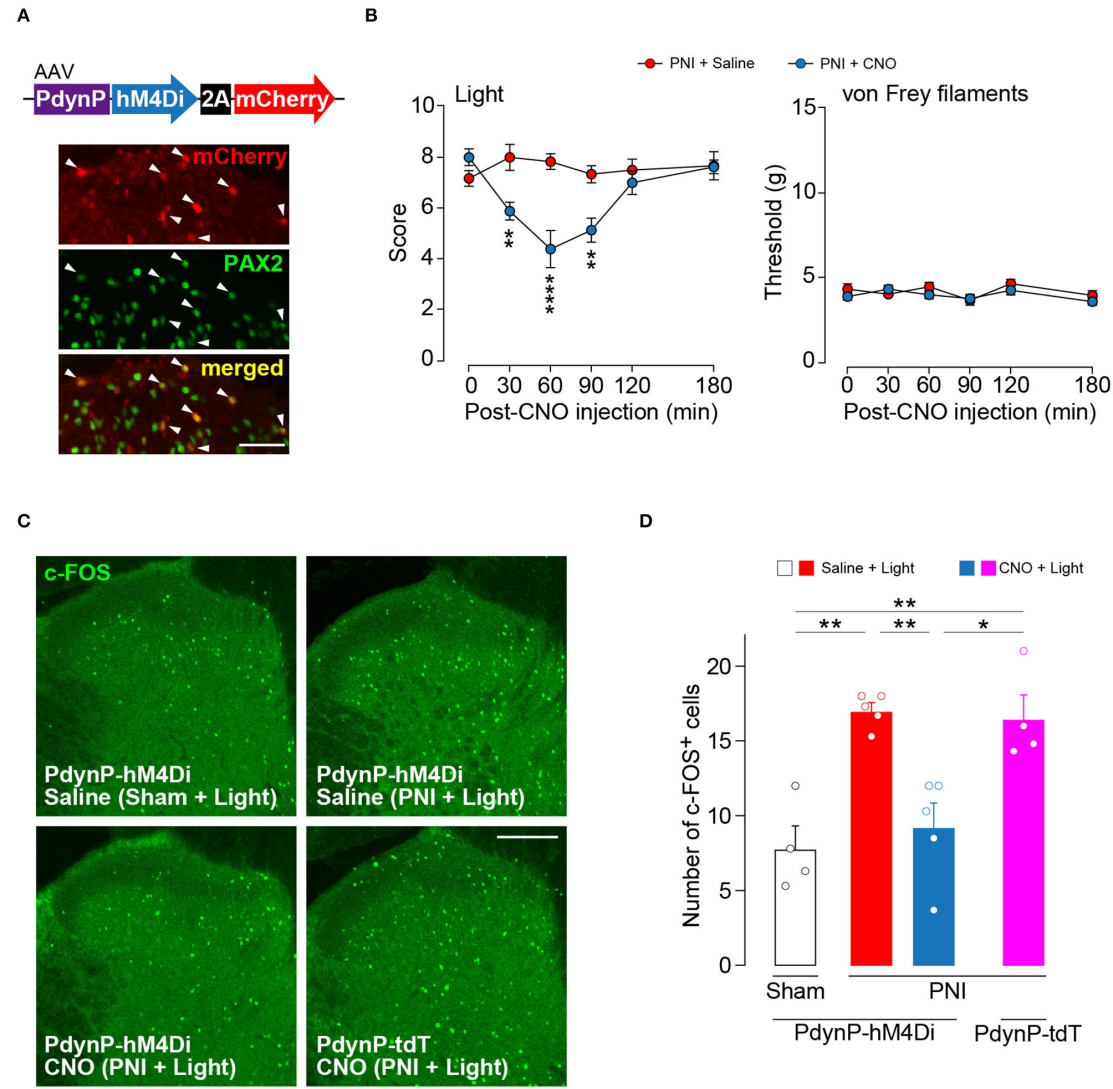
Optogenetic Aβ fiber-induced pain-like behavior after peripheral nerve injury (PNI) was attenuated by silencing AAV-PdynP^+^ neurons in rats. **(A)** Colocalization of mCherry and PAX2 (green) in the L4 spinal dorsal horn (SDH) at 4 weeks after intra-SDH microinjection of AAV-PdynP-hM4Di-2A-mCherry (indicated by arrowheads). Scale bar, 50 μm. **(B)** Paw withdrawal behaviors from light and von Frey filaments before and after injection with the agonist CNO (1 mg/kg) or saline injection into W-TChR2V4 rats with PNI (*n* = 6–8 rats). **P < 0.01, and ****P < 0.0001, two-way repeated measures ANOVA with *post-hoc* Bonferroni multiple comparison test. **(C,D)** Immunofluorescence **(C)** and number **(D)** of c-FOS^+^ neurons in laminae I–II of the L4 SDH of W-TChR2V4 rats with light stimulation 2 weeks after sham operation or PNI (*n* = 4–5 rats). Scale bar, 200 μm. *P < 0.05, and **P < 0.01, one-way ANOVA with *post-hoc* Bonferroni multiple comparison test. Data shown as mean ± SEM.

Our findings suggest that acute silencing of AAV-PdynP^+^ SDH neurons could suppress optogenetic Aβ fiber-derived neuropathic allodynia-like behavior. To examine this, we employed a chemogenetic approach using the inhibitory DREADD hM4Di that enables the silencing of neurons by its agonist clozapine-N-oxide (CNO) (Roth, 2016). In WT rats with an intra-SDH injection of AAV-PdynP-hM4Di-2A-mCherry (Figure 5A), hM4Di^+^ neurons (mCherry^+^) abounded in superficial laminae of the SDH, and many of which were positive to PAX2. In hM4Di-expressing W-TChR2V4 rats at 2 weeks post-PNI, i.p. injection of CNO significantly decreased paw withdrawal behavior from light stimulation of Aβ fibers, but not from von Frey filaments (Figure 5B). Furthermore, the number of neurons positive for c-FOS (a neural activity marker) evoked by optical Aβ fiber stimulation was increased in superficial laminae of the SDH after PNI (upper right in Figure 5C and the red bar in Figure 5D), but this was also suppressed in hM4Di-expressing rats given CNO injection (lower left in Figure 5C and the blue bar in Figure 5D). The number of c-FOS^+^ neurons in the ipsilateral SDH was not affected via CNO administration alone (lower right in Figure 5C and the pink bar in Figure 5D). These findings suggest that functional inhibition of AAV-PdynP^+^ neurons after PNI suppresses optogenetic Aβ fiber-induced neuropathic allodynia-like behavior.

## Discussion

In this study, we showed that intra-SDH injection of a designed AAV-PdynP vector captured a subset of SDH interneurons. The majority of these neurons were located in the superficial layers including lamina I and were inhibitory. Their laminar distribution and the proportion with and without PAX2 are consistent with a previous report of SDH neurons immunolabeled with an antibody for preprodynorphin in rats (Sardella et al., 2011). Our study also confirmed that AAV-PdynP^+^ neurons expressed PDYN, suggesting that the expression of the incorporated gene into the vector was not non-specific but was regulated by the activity of the *Pdyn* promoter. However, AAV-PdynP injected into the SDH could not capture all PDNY-expressing neurons, which was likely related to the length of the *Pdyn* promoter we used (~2 kbp: almost the maximum size that can be incorporated into the AAV vector under our experimental conditions). Nevertheless, the cell ablation approach using the DTX-DTR system revealed that these AAV-PdynP-captured SDH neurons critically contribute to touch-sensing optogenetic Aβ fiber-evoked neuropathic behavioral hypersensitivity and aversive response (resembling mechanical allodynia) without affecting normal behavioral responses evoked by the optical stimulation of Aβ fibers, as well as nociceptive mechanical force and heat. Furthermore, acute chemogenetic silencing of these neurons alleviated the optogenetic Aβ fiber-evoked hypersensitivity that developed after PNI, implicating their ongoing pivotal role in neuropathic mechanical allodynia-like behavior. Notably, ablation of the AAV-PdynP^+^ neurons did not affect the PNI-induced hypersensitivity to mechanical stimulation by von Frey filaments. Previous reports demonstrated that the withdrawal threshold from von Frey filaments after PNI was suppressed by silencing or ablating nociceptive C or Aδ fibers (Boada et al., 2014; Iyer et al., 2014; Daou et al., 2016), implying the filaments activate not only LTMRs but also nociceptors (Tsuda, 2019). In addition, the difference in the efficacy of morphine to suppress the hypersensitivity has also been shown for these two stimuli (Tashima et al., 2018). Since W-TChR2V4 rats express ChR2 in Aβ fibers associated with the touch sensors Merkel cells and Meissner corpuscles (Ji et al., 2012), it seems that the AAV-PdynP-captured SDH interneurons are selectively involved in touch-sensing optogenetic Aβ fiber-evoked neuropathic allodynia-like behavioral responses.

While this study highlights the ability of AAV-PdynP^+^ neurons to produce touch-evoked allodynia-like behavior in rats, it should be noted that the role of *Pdyn*-expressing neurons is inconsistent. It has been shown that BHLHB5-deficient mice without *Pdyn*-expressing neurons exhibit normal behavioral responses to mechanical stimuli (although these mice do exhibit spontaneous scratching behavior) (Ross et al., 2010; Kardon et al., 2014). In contrast, another study utilizing an intersectional strategy combining *Pdyn-Cre* mice with other mouse lines to selectively manipulate *Pdyn*-lineage neurons showed that mice with ablation of these neurons become hypersensitive to mechanical stimulation from both von Frey filaments and a paintbrush (Duan et al., 2014). Acute silencing of adult *Pdyn*−*Cre*^+^ SDH neurons also elicits similar hypersensitivities (Zhang et al., 2018), but it has also been shown that activation of these neurons produces mechanical hypersensitivity (Huang et al., 2018). The reason for the differences among these studies including ours is unclear, but there are some possibilities. First, it might be related to the different species involved. The laminar distribution of AAV-PdynP^+^ neurons in rats and *Pdyn*-lineage neurons in *Pdyn-Cre* mice are different. Indeed, AAV-PdynP^+^ neurons were found to be mainly localized in lamina I, but *Pdyn*-lineage neurons were distributed not only in lamina I but also in the deeper laminae (Duan et al., 2014). In addition, preprodynorphin^+^ excitatory neurons in the SDH were highly localized in the medial part in mice (Huang et al., 2018), but such a spatial pattern was not observed in rats (Sardella et al., 2011). Second, AAV-PdynP-captured SDH neurons whose *Pdyn* promoter is active in adults, while the *Pdyn-Cre* mouse line can capture *Pdyn*-lineage neurons whose *Pdyn* promoter-dependent Cre expression occurs at any stage in the lifespan (including development). Third, given that *Pdyn*-expressing SDH neurons are heterogeneous (Serafin et al., 2021), the proportion and specificity of *Pdyn*-expressing neurons that are manipulated may also be related to the differences. In our study, the AAV-PdynP captured a portion of the PDYN-expressing neurons located in the injected segments. In addition, among the AAV-PdynP^+^ neurons in the SDH, the majority of neurons were inhibitory, but the remaining neurons seemed to be excitatory. Thus, these possibilities described above may account in part for the different behavioral outcomes by the manipulation of SDH neurons with PDYN expression or active *Pdyn* promoter. Nevertheless, it is worth noting that the subset of SDH neurons captured by the AAV-PdynP may be an important clue to elucidate the mechanistic underpinnings of neuropathic mechanical allodynia-like behavioral responses in rats.

The pathologically altered function of AAV-PdynP^+^ neurons after PNI appears to be a key phenomenon for neuropathic mechanical allodynia-like behavior. This is strongly supported by a correlation between the electrophysiological data (elevated RMPs of these neurons) and the behavior (optogenetic Aβ fiber-evoked withdrawal response) obtained from the individual rats that were either sham-operated or had PNI and by a reversal effect from AAV-PdynP^+^ neuron silencing in neuropathic allodynia-like behavior. Considering that AAV-PdynP^+^ neurons normally did not fire by optical Aβ fiber stimulation but did after PNI, it seems that PNI opens a gate for Aβ fiber signals to AAV-PdynP^+^ neurons. Enhanced excitatory polysynaptic inputs from optogenetic Aβ fibers and elevated RMPs in these neurons render these neurons sensitive to optogenetic Aβ fiber-derived signals, which in turn produce APs. The transition from silent to functional synapse has been shown to involve α-amino-3-hydroxy-5-methyl-4-isoxalepropionate (AMPA) receptors. It was previously reported that after PNI, the expression and cell-surface recruitment of AMPA receptors is increased in SDH neurons (Harris et al., 1996), which enhances AMPA receptor-mediated synaptic responses to innocuous stimuli (Li and Zhuo, 1998). Furthermore, voltage-gated K^+^ channels or leak K^+^ current through two-pore domain K^+^ channels influence RMPs (Wulff et al., 2009; Blankenship et al., 2013). Further, it is known that astrocytes are activated after PNI (Tsuda et al., 2011; Kohro et al., 2015) and are involved in the clearance of extracellular K^+^ (Bellot-Saez et al., 2021). Thus, activated astrocytes may be involved in the hyperexcitability of AAV-PdynP^+^ neurons after PNI via a change in extracellular K^+^ concentration. The pathologically gained excitation of AAV-PdynP^+^ neurons is considered to alter somatosensory information processing leading to the conversion of Aβ fiber-derived signal to become nociceptive. Given that AAV-PdynP^+^ neurons are mostly inhibitory, a circuit model could be considered: AAV-PdynP^+^ neurons negatively control certain inhibitory interneurons, which could, in turn, result in the activation of superficial neurons involved in transmitting and processing nociceptive information. Accordingly, PNI increased the optogenetic Aβ fiber-evoked c-FOS^+^ neurons in the superficial laminae, which was reduced by AAV-PdynP^+^ neuron silencing. However, more detailed pathways from AAV-PdynP^+^ neurons to brain-projecting nociceptive neurons need further investigations.

In summary, this study demonstrated that a subset of SDH inhibitory interneurons captured by AAV-PdynP located mainly in the superficial laminae became excitable to optical Aβ fiber stimulation after PNI. The optogenetic Aβ fiber-mediated neuropathic allodynia-like behavioral responses were attenuated by ablation or silencing of AAV-PdynP-captured SDH neurons, suggesting that these neurons critically contribute to optogenetic Aβ fiber-induced neuropathic allodynia-like behavior. Thus, AAV-PdynP-captured SDH neurons may be potential targets for elucidating the mechanistic underpinnings of Aβ fiber-dependent neuropathic allodynia and for treating neuropathic pain.

## Data Availability Statement

The original contributions presented in the study are included in the article/Supplementary Material, further inquiries can be directed to the corresponding author.

## Ethics Statement

The animal study was reviewed and approved by Kyushu University.

## Author Contributions

TI, YY, and DS designed experiments, performed experiments, analyzed the data, and wrote the manuscript. RT designed all AAV vectors, performed experiments, and analyzed the data. HT-S and KK assisted experiments. KY provided comments on experiments. MT conceived and initiated this project, designed experiments, supervised the overall project, and wrote the manuscript. All authors read and approved the submitted version.

## Funding

This work was supported by the JSPS KAKENHI Grant Numbers JP19H05658 and JP20H05900 (MT), and by the Platform Project for Supporting Drug Discovery and Life Science Research [Basis for Supporting Innovative Drug Discovery and Life Science Research (BINDS)] from AMED under Grant Number JP21am0101091 (MT).

## Conflict of Interest

The authors declare that the research was conducted in the absence of any commercial or financial relationships that could be construed as a potential conflict of interest.

## Publisher's Note

All claims expressed in this article are solely those of the authors and do not necessarily represent those of their affiliated organizations, or those of the publisher, the editors and the reviewers. Any product that may be evaluated in this article, or claim that may be made by its manufacturer, is not guaranteed or endorsed by the publisher.
