# Supplementary material for: Selective Involvement of a Subset of Spinal Dorsal Horn Neurons Operated by a Prodynorphin Promoter in Aβ Fiber-Mediated Neuropathic Allodynia-Like Behavioral Responses in Rats
Source: Front Mol Neurosci. 2022 Jun 23;15:911122. doi: 10.3389/fnmol.2022.911122 (PMC9260077; doi:10.3389/fnmol.2022.911122)
Supplement: Supplementary file 1 [file Table_1.DOCX]

**Supplemental Table 1**

|  | Sham | PNI | *P* value |
| --- | --- | --- | --- |
| Input resistance (MΩ) | 393.37 ± 99.24 | 426.39 ± 70.43 | 0.4002 |
| Membrane capacitance (pF) | 44.56 ± 5.20 | 44.99 ± 4.66 | 0.7802 |

**Supplemental Table 1. Electrophysiological properties of AAV-PdynP^+^ neurons in W-TChR2V4 rats with sham or PNI**

Input resistance and membrane capacitance of AAV-PdynP^+^ neurons with sham or PNI were quantified (n = 9 neurons tested from 6 W-TChR2V4 rats with sham operation, n = 10 neurons tested from 8 W-TChR2V4 rats with PNI). Mann-Whitney *U* test. Data show the mean ± sem.
